# Artificial intelligence in clinical endoscopy: Insights in the field of videomics

**DOI:** 10.3389/fsurg.2022.933297

**Published:** 2022-09-12

**Authors:** Alberto Paderno, Francesca Gennarini, Alessandra Sordi, Claudia Montenegro, Davide Lancini, Francesca Pia Villani, Sara Moccia, Cesare Piazza

**Affiliations:** ^1^ Unit of Otorhinolaryngology—Head and Neck Surgery, ASST Spedali Civili of Brescia, Brescia, Italy; ^2^ Department of Medical and Surgical Specialties, Radiological Sciences, and Public Health, School of Medicine, University of Brescia, Brescia, Italy; ^3^ The BioRobotics Institute, Scuola Superiore Sant’Anna, Pisa, Italy; ^4^ Department of Excellence in Robotics and AI, Scuola Superiore Sant’Anna, Pisa, Italy

**Keywords:** artificial intelligence, machine learning, videomics, endoscopy, neural networks

## Abstract

Artificial intelligence is being increasingly seen as a useful tool in medicine. Specifically, these technologies have the objective to extract insights from complex datasets that cannot easily be analyzed by conventional statistical methods. While promising results have been obtained for various -omics datasets, radiological images, and histopathologic slides, analysis of videoendoscopic frames still represents a major challenge. In this context, videomics represents a burgeoning field wherein several methods of computer vision are systematically used to organize unstructured data from frames obtained during diagnostic videoendoscopy. Recent studies have focused on five broad tasks with increasing complexity: quality assessment of endoscopic images, classification of pathologic and nonpathologic frames, detection of lesions inside frames, segmentation of pathologic lesions, and in-depth characterization of neoplastic lesions. Herein, we present a broad overview of the field, with a focus on conceptual key points and future perspectives.

## Introduction

Use of artificial intelligence (AI) is currently increasing in every field of medicine, progressively encompassing the entire patient care process, from embryo selection to survival prediction (1). Machine learning (ML), a branch of AI, has the objective of automatically extracting actionable insights from complex and large datasets that cannot (or are hard to) be effectively analyzed by conventional statistical methods or human intuition. ML algorithms have been designed to tackle the variability of “-omics” datasets (e.g., genomics, epigenomics, transcriptomics, and proteomics), and unstructured data as radiologic images (radiomics), histopathology (pathomics), and surgical or videoendoscopic images (videomics) (2). In this area, ML is being applied to identify disease patterns and predict specific characteristics that may assist clinicians in diagnosis, therapeutic management, and follow-up.

While promising results have been obtained for -omics datasets and radiological and histopathologic images, analysis of videoendoscopic frames still represents a challenge. In this context, videomics represents a burgeoning field wherein several methods of computer vision are systematically used to organize the unstructured data of frames obtained during diagnostic videoendoscopy. These applications are still in the early stage of development, especially in the field of otolaryngology—head and neck surgery, where the term videomics was first introduced (2). In particular, one of the main limits in developing robust and automatic ML algorithms that can be translated in the clinical practice is represented by the paucity of annotated datasets to train algorithms.

## Aims of videomics

Diagnostic endoscopy is an essential component in the assessment of the upper aerodigestive tract (UADT) and is a cornerstone as a first-line diagnostic tool, especially after the introduction of the “bioendoscopy” concept (3). The introduction of videoendoscopy significantly improved this field by the development of high-quality video recording, image magnification, high-definition visualization, and advanced optical filters such as Narrow Band Imaging (NBI), Storz Professional Image Enhancement System (SPIES or Image 1S), and I-Scan. These nuances, together with the constant advancement in ML, have opened new possibilities for image analysis in a computer vision-oriented approach. Here, deep learning (DL), a branch of ML, is playing a paramount role.

**Figure 1:**
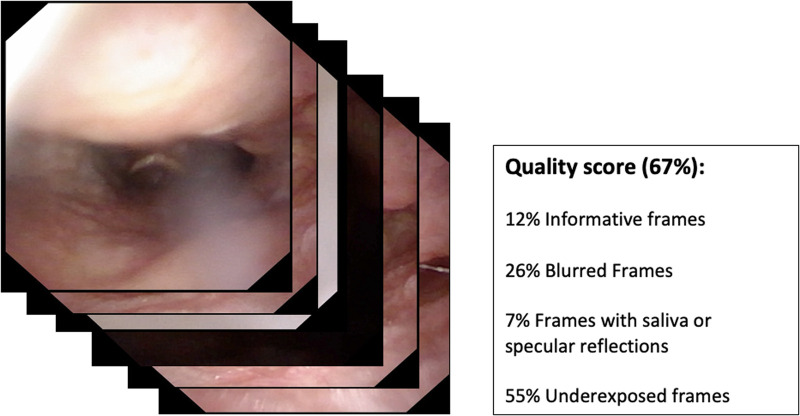
Depiction of the potential input and output of a quality assessment algorithm.

**Figure 2:**
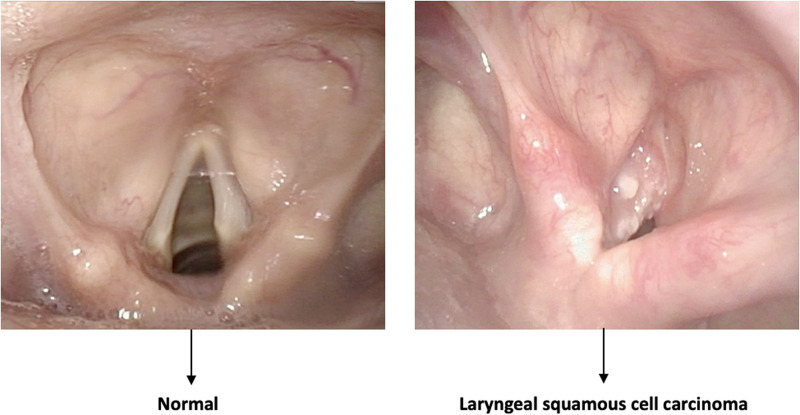
Example of a classification task. The algorithm distinguishes between normal and pathologic frames without identifying the area involved by the disease.

**Figure 3:**
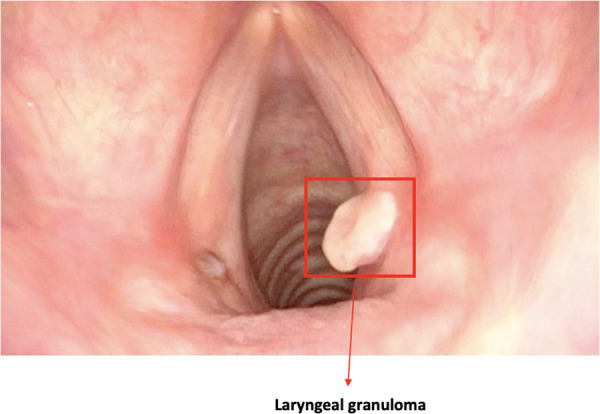
Image showing a bounding box localizing a laryngeal lesion. This is the typical output of detection algorithms.

**Figure 4:**
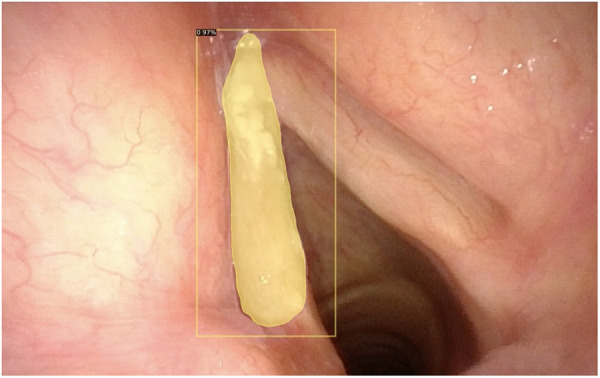
Automatic segmentation of a laryngeal lesion provided by a convolutional neural network after adequate training and optimization.

**Figure 5:**
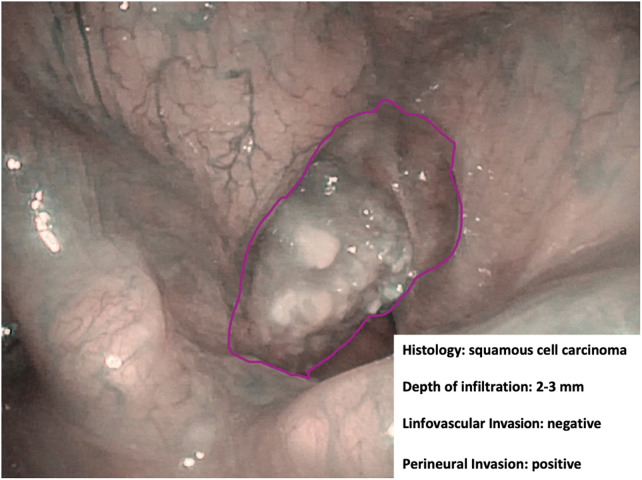
Endoscopic NBI frame showing an example of adjunctive data drawn from in-depth characterization by hypothetical machine learning algorithms.

In the field of supervised learning, when provided with both the “problem” (i.e., unlabeled videoendoscopic frame) and the “solution” (i.e., annotated frame or “ground truth”), DL algorithms iteratively learn their internal parameters (i.e., weights) to progressively improve diagnostic performance and specialize on a given objective. In this field, recent studies have focused on five broad tasks with increasing complexity and computational load, which can be summarized as follows:

• Quality assessment of endoscopic images (Figure 1);
• Classification of pathologic and nonpathologic frames (Figure 2);
• Detection of lesions inside frames (Figure 3);
• Segmentation of pathologic lesions (Figure 4);
• In-depth characterization of neoplastic lesions (Figure 5).

In this area, a stepwise approach has the potential to make use of incremental refinements of algorithms and develop functional “minimal viable products” that can be introduced in clinical practice as early as possible, even without the full suite of the above-mentioned applications. This is especially true considering that, as mentioned, the main limiting factor in this field is the paucity of large dedicated datasets that are usable for training. Gómez et al. (4) initially addressed this issue in the field of high-speed laryngeal videoendoscopy by collecting and publishing the Benchmark for Automatic Glottis Segmentation (BAGLS) multihospital glottis segmentation dataset. However, with the progressive expansion of the available training images, it will be possible to tackle increasingly complex challenges.

Furthermore, the application of transfer learning techniques may significantly improve algorithm training and reduce the number of images needed to achieve optimal performance. Transfer learning consists in pretraining the algorithm with images that are not directly correlated to the task to be explored, but which have certain similarities with the target dataset. For example, the weights of convolutional neural networks (CNNs) for endoscopic image analysis are often initialized with weights obtained by training CNNs on natural images from everyday objects (i.e., ImageNet dataset). This allows CNNs to detect generic low-level features (e.g., corners, edges). Pretraining with endoscopic images from a different anatomic site may provide an adjunctive advantage, especially in small datasets. CNNs are then fine-tuned to slightly adjust their parameters using endoscopic images.

A potential approach to address the low number of manually annotated images is offered by unsupervised and self-supervised learning. Unlike supervised learning, which is biased toward how it is being supervised, unsupervised learning derives insights directly from the data itself, groups the data, and helps make data-driven decisions without external biases (5). This approach may be particularly useful to cluster endoscopic frames into different categories (e.g., low visibility vs. good visibility) to help the clinician’s assessment. On the other hand, self-supervised learning takes advantage of unlabeled images of the same pathology but captured from different views to significantly enhance the performance of pretraining. However, these options still need to be fully explored in the field of UADT endoscopy (6).

### Quality assessment

The first step in which AI can be effectively applied to diagnostic videoendoscopies is their quality control. In fact, in every examination, the majority of videoendoscopic frames are not diagnostic due to the presence of technical or patient-related factors that limit visualization. These factors, in the field of UADT evaluation, are mainly represented by repeated swallowing, gag reflex, secretions, blurring of the camera, specular reflections, and over- and underexposure. Automatic identification and classification of these issues can be of help in real-time determination of the quality of an endoscopic examination and may allow automatic detection of the most significant frames in a given recording.

In this field, Patrini et al. (7) developed a ML-based strategy for automatic selection of informative videolaryngoscopic frames. This approach resulted in a recall (i.e., true positive rate = true positives over true positives and false negatives) of 0.97 when classifying informative vs. uninformative frames (i.e., blurred, with saliva or specular reflections, and underexposed) with support vector machines (SVM) (i.e., conventional ML algorithms) and of 0.98 with a CNN-based classification. Furthermore, their work demonstrated the potential of transfer learning in medical image analysis.

As a proof of concept, recent advances in the field of gastrointestinal endoscopy have led to the development of a fully automatic framework that can detect and classify different artifacts, segment artifact instances, provide a quality score for each frame, and restore partially corrupted frames (8).

### Classification

Classification is a typical task in the field of DL, distinguishing between normal and pathological mucosa. Here, the objective is not to localize or finely characterize a particular lesion, but rather to distinguish entire frames into different classes, usually pathologic vs. nonpathologic.

In this field, He et al. (9) applied CNN to interpret images of laryngeal squamous cell carcinoma using static NBI frames to determine whether a lesion was benign or malignant. The model reached an accuracy of 90.6%, a sensitivity of 88.8%, and a specificity of 92.2%. Furthermore, the authors demonstrated that the accuracy of the CNN model was higher than that of human experts. A similar approach was described by Esmaeili et al. (10), training a CNN for the automatic classification of NBI images into benign and malignant. A pretrained ResNet50 architecture was adopted, and three experiments with several models were generated and validated. The model showed a striking diagnostic performance and achieved a testing accuracy of 0.83.

Considering multiple classification groups, Zhao et al. (11) proposed a four class-system of vocal cord targets (i.e., normal mucosa, polyp, keratinization, and carcinoma), and a laryngoscopy dataset was divided into “urgent” (keratinization, carcinoma) and “nonurgent” (normal mucosa, polyp) cases. An overall accuracy of 80.2%, an F1 score (i.e., the harmonic mean of the precision and recall, a measure of accuracy) of 0.78, and an area under the curve (AUC) of 0.96 were achieved. The proposed method delivered high classification performance of normal mucosa, polyps, and carcinoma in extremely quick time.

Other studies (12, 13) have employed ML to classify pharyngo-laryngeal benign lesions during videoendoscopy, demonstrating notable results. A preliminary attempt was described in 2014 by Huang et al. (12), who proposed an automatic system aimed at recognizing the dynamic image of the glottis and classifying different vocal fold disorders (“normal vocal fold,” “vocal fold paralysis,” “vocal fold polyp,” and “vocal fold cyst”). This study used an SVM classifier and reached an accuracy of 98.7%. However, the patterns to be classified did not include dysplasia or malignancy. Dunham et al. (13) proposed the concept of “optical biopsy” (already introduced by our group before implementing AI applications in videomics) (14) using CNN technology. The first objective was to classify endoscopic images into one of five benign classes (normal mucosa, nodules, papilloma, polyps, and webs). The second was, using a binary classifier, to distinguish malignant/premalignant from benign lesions. The overall accuracy for the multiclass benign vocal fold lesion classifier was 80.8%, while the binary test achieved an overall accuracy of 93%.

Different authors (15, 16) also demonstrated the feasibility of classifying oropharyngeal and oral cavity (OC) lesions using ML technology. For the oropharynx (OP), Mascharak et al. (15) used a naive Bayesian classifier (color and texture) to demonstrate the value of NBI imaging instead of white light (WL) videoendoscopy, which added more definition to tumor margins and highlighted submucosal vascularization. Fivefold cross-validation provided an AUC of over 80% for NBI and under 55% for WL endoscopy models (*p* < 0.001).

In the oral cavity, in 2018, Song et al. (16), employing CNNs, proposed a low-cost, smartphone-based, automatic image classification system. The authors collected data from 190 patients across several centers in India to detect oral dysplasia and malignancy using a dual-mode image analysis with WL and autofluorescence (AF). The study compared the accuracy of the single- (WL or AF) and dual-mode (WL and AF) image analysis, demonstrating that the latter had a better diagnostic performance. The final model reached an accuracy of 87%, a sensitivity of 85%, and a specificity of 89%.

### Detection

Lesion detection remains the main objective of DL-based strategies in contemporary clinical videoendoscopy. Different authors have described the potential of CNN in the detection of cancer, premalignant lesions, benign lesions, and normal tissue. In this setting, algorithms are constantly being improved that better conform to specific tasks or subsites.

Inaba et al. (17) trained a CNN-based algorithm (RetinaNet) to detect superficial laryngo-pharyngeal cancer. To evaluate diagnostic accuracy, 400 pathologic images and 800 of normal mucosa were collected, reaching an accuracy, sensitivity, and specificity of 97%, 95%, and 98%, respectively. The definition of correct diagnosis was set with an intersection over union (IoU) (i.e., the measure of overlap between prediction and ground truth) >0.4. Interestingly, the authors showed a direct correlation between the algorithm diagnostic performance and the number of images used for training. This is a not surprising outcome and clearly highlights the importance of training data, both in quantitative and in qualitative terms, during the training phase of an algorithm. In fact, to date, the low number and small size of the available medically oriented datasets are the real bottlenecks that limit the development of clinically relevant computer vision algorithms. A similar approach was described by Xiong et al. (18), who developed a CNN-based diagnostic system using videoendoscopic images of laryngeal cancer, premalignant lesions, benign lesions, and normal tissue. The CNN detected lesions with an accuracy of 87%, a sensitivity of 73%, a specificity of 92%, and an AUC of 92%. Moreover, the results were comparable to those obtained by a human expert with 20 years of experience.

With regard to real-time detection, Matava et al. (19) and Azam et al. (20) developed CNN algorithms that were applied in real time during videoendoscopy and which aimed at identifying, on the one hand, normal airway anatomy and, on the other hand, UADT lesions. Using this type of approach, DL may be a useful complementary tool for clinicians in endoscopic examinations, progressively implementing the concept of human–computer collaboration. In detail, Matava et al. (19) compared the predictive performance of three models (ResNet, Inception, and MobileNet) in the identification of normal components of laryngeal and tracheal airway anatomy. ResNet and Inception achieved a specificity of 0.98 and 0.97 and a sensitivity of 0.89 and 0.86, respectively. Finally, Azam et al. (20) identified a CNN model for real-time laryngeal cancer detection in WL and NBI videoendoscopies. The dataset, consisting of 219 patients, was tested with an algorithm that achieved 0.66 precision (i.e., positive predictive value = true positives over true and false positives), 0.62 recall, and 0.63 mean average precision with an IoU > 0.5. In addition, the model ran with an average computation time per video frame of 0.026 s.

### Segmentation

Automated segmentation of anatomical structures in medical image analysis is a prerequisite for autonomous diagnosis and represents one of the most complex tasks in the field of computer vision. In this case, the algorithm does not only need to detect lesions but also need to automatically delineate their margins. Recent CNN-based methods have demonstrated remarkable results and are well-suited for such a complex task.

During transoral laser microsurgery, a seven-class (void, vocal folds, other tissue, glottic space, pathology, surgical tools, and tracheal tube) dataset was trained by Laves et al. (21) using a CNN-based algorithm. Different CNN architectures were investigated, and a weighted average ensemble network of UNet and ErfNet (two of the most commonly used CNNs) turned out to be the best suited for laryngeal segmentation, with a mean IoU of 84.7%. Advances in ML and computer vision have led to the development of methods for accurate and efficient real-time segmentation. Paderno et al. (22) explored the use of fully CNNs for real-time segmentation of squamous cell cancer in videoendoscopies of the OC and OP. In this work, the authors compared different architectures and detailed their diagnostic performance and inference time, demonstrating their significant potential and the possibility of achieving real-time segmentation. However, for the first time, they suggested that highly heterogeneous subsites such as those encountered in the OC may have inferior results when compared with more structurally homogeneous areas such as the OP. This is in line with what was previously observed when applying bioendoscopic tools alone in a non-AI environment by Piazza et al. (14) and is possibly related to the larger epithelial differentiation within the OC vs. the OP and to specific limits related to oral examination (the presence of light artifacts and confounders such as tongue blade, teeth, or dentures).

When dealing with laryngeal lesions, Fehling et al. (23) explored the possibility of achieving a fully automated segmentation of the glottic area and vocal fold tissue using a CNN in high-speed laryngeal videos. The algorithm obtained a Dice similarity coefficient (i.e., the measure that evaluates the intersection of the two regions as a ratio to the total area of them both) of 0.85 for the glottis, 0.91 for the right, and 0.90 for the left vocal fold. Furthermore, the results revealed that, in both pathologic and healthy subjects, the automatic segmentation accuracy obtained was comparable or even superior to manual segmentation.

Generally, laryngo-pharyngeal lesions are those more frequently examined when measuring the role of automatic analysis by ML. In fact, only limited studies on nasopharyngeal disease differentiation have been performed on the basis of endoscopic images. For example, Li et al. (24) proposed a method to segment nasopharyngeal malignancies in endoscopic images based on DL. The final model reached an accuracy of 88.0%.

Finally, DL proved to be a promising addition to the field of endoscopic laryngeal high-speed videos. In clinical practice, the previous lack of dedicated software to analyze the data obtained resulted in a purely subjective assessment of the symmetry of vocal fold movement and oscillation. The development of easy-to-use DL-based systems that are capable of automatic glottal detection and midline segmentation allowed obtaining objective functional data without the need for manual or semiautomatic annotation as previously described, among others, by Piazza et al. (25), thus significantly simplifying the process. These results were obtained through an organized and stepwise approach headed by the Erlangen research group that achieved high-fidelity automatic segmentation of the glottis (23) and glottal midline (26) as well as extraction of relevant functional parameters (27). Thanks to these preliminary data, a DL-enhanced software tool for laryngeal dynamics analysis was developed (28). This software provides 79 unique quantitative analysis parameters for video- and audio-based signals, and most of these have already been shown to reflect voice disorders, highlighting its clinical importance.

### In-depth characterization

All the previously described tasks aim to provide an accurate definition of a given lesion, classifying it according to its nature, defining its location in the frame, and delineating its margins (with possible future roles in real-time definition of resection margins during a surgical procedure). However, all these objectives reproduce only what is generally achieved by an expert clinician and do not try to overcome the limits of human perception, even though their future implementation within a telemedicine environment would represent a large step toward more homogeneous diagnostic opportunities.

However, there is already indirect evidence that pattern recognition capabilities of novel AI systems may allow finding a correlation between the endoscopic appearance of a given lesion and its finer characteristics. Among these, depth of infiltration, so far investigable only by radiologic imaging or histopathologic evaluation (29), plays a remarkable role in the prognostication of OC cancer and has fueled great interest in the possibility of speeding up its definition by AI tools applied to videomics. Identification through videomics of other tumor characteristics, such as histopathological risk factors (e.g., perineural and lymphovascular invasion), viral status (human papilloma and Epstein–Barr viruses), and genomic markers, is definitively more ambitious but already within the reach of similar approaches like radiomics and pathomics. Bridges connecting all these sources of information would be of great help in the near future to build up sharable profiling of tumors and their microenvironment.

Recent studies in the gastrointestinal tract, for example, have provided the proof of concept of this hypothesis and demonstrated that CNNs can differentiate between early and deeply infiltrating gastric cancer (30). Nakahira et al. (31) further confirmed the potential of this approach by showing that CNN was able to correctly stratify the risk of gastric tumor development by analyzing the non-neoplastic mucosa at videoendoscopy.

## Future perspectives

The introduction of computer vision in UADT endoscopy is still in its infancy and further steps will need to be taken before reaching widespread application. In this view, the first step outside of purely research-driven applications will be the use of ML algorithms for human–computer collaboration. Dedicated algorithms can assist in every step of the endoscopic diagnostic approach, from quality assurance, effective storage and video classification, to risk determination, histologic definition, margins evaluation, and in-depth lesion profiling. As previously stated, this will be a stepwise approach that will start from easier tasks (i.e., quality assurance) and will progress toward more complex and more clinically relevant objectives. The ideal outcome will be to achieve accurate lesion characterization in terms of histologic nature, margins, and biologic characteristics and to be able to fully and objectively integrate these insights with data from other types of examinations (e.g., radiology, molecular biology, and histopathology).

Morphologic image analysis is the main field in which videomics is evolving in the context of clinical endoscopy. However, other more innovative aspects can be assessed by taking advantage of current computer vision technologies. A particularly interesting feature in otolaryngology is vocal fold motility; in fact, an objective evaluation of this variable can be extremely helpful in both assessment of functional deficits and in the precise staging of neoplastic disease of the glottis. This is especially true when considering that the AJCC/UICC TNM classification (32) of laryngeal cancer relies on purely subjective definitions of “normal vocal cord mobility,” “impaired vocal cord mobility,” and “vocal cord fixation” for the categorization of T1, T2, and T3 glottic tumors, respectively.

In this field, Adamian et al. (33) recently developed an open-source computer vision tool for automated vocal fold tracking from videoendoscopies that is capable of estimating the anterior angle between vocal folds of subjects with normal mobility and those with unilateral vocal fold paralysis. The authors demonstrated the possibility of identifying patients with vocal fold palsy by assessing the angle of maximal glottic opening (49° vs. 69°; *p* < 0.001). In particular, an angle of maximum opening <58.6° was predictive of paralysis with a sensitivity and specificity of 0.85. Notwithstanding, this approach places significant limits on the evaluation of reduced mobility due to neoplastic involvement since it relies on the identification of the free margin of vocal folds, which is often altered by glottic tumors. However, the development of alternative strategies is providing valuable outcomes in such a task.

Finally, novel surgical technologies such as transoral robotic (34) and exoscopic surgery (35) rely on digital video acquisition of a large amount of data and will potentially extend the applications of videomics to the intraoperative setting of quality and safety control as well as didactic proficiency. This is especially interesting considering the urgent need for more extensive training and collaborative datasets that will enable better refinement of ML algorithms, coming not only from diagnostic instrumentation but also from surgical robots and exoscopic tools.
